# Mechanisms of the JNK/p38 MAPK signaling pathway in drug resistance in ovarian cancer

**DOI:** 10.3389/fonc.2025.1533352

**Published:** 2025-04-24

**Authors:** Yu-Ting Ma, Chan Li, Ying Shen, Wan-Hui You, Ming-Xuan Han, Yi-Fan Mu, Feng-Juan Han

**Affiliations:** ^1^ Department of Obstetrics and Gynecology, Heilongjiang University of Chinese Medicine, Harbin, Heilongjiang, China; ^2^ Department of Obstetrics and Gynecology, First Affiliated Hospital, Heilongjiang University of Chinese Medicine, Harbin, Heilongjiang, China

**Keywords:** ovarian cancer, drug-resistant, JNK, p38, MAPK, apoptosis, natural compounds

## Abstract

Ovarian cancer (OC) is the most lethal malignancy in the female reproductive system, and chemotherapy drug resistance is the main cause of treatment failure. The Mitogen-Activated Protein Kinases (MAPK) pathway plays a pivotal role in regulating cell proliferation, migration, and invasive capacity in response to extracellular stimuli. This review focuses on the mechanisms and therapeutic strategies related to the JNK/p38 MAPK signaling pathway in OC resistance. The JNK/p38 MAPK pathway plays a dual role in OC chemoresistance. This review examines its role in mediating OC treatment resistance by exploring the mechanisms of action of the JNK/p38 MAPK signaling pathway, particularly its involvement in several key biological processes, including apoptosis, autophagy, DNA damage response, the tumor microenvironment (TME), and drug efflux. Additionally, the review investigates the timing of activation of this pathway and its crosstalk with other signaling pathways such as PI3K/AKT and NF-κB. Targeting JNK/p38 MAPK signaling has shown promise in reversing chemoresistance, with several inhibitors and natural compounds demonstrating potential in preclinical studies. Regulating JNK/p38 MAPK may transform what was once a terminal obstacle into a manageable challenge for OC patients with chemotherapy resistance, ultimately improving survival and quality of life.

## 1 Introduction

Ovarian cancer (OC) is the gynecologic malignancy with the highest mortality rate, with approximately 19,680 new cases of OC diagnosed in the United States in 2024 (1). OC accounts for 5.1% of all female cancer deaths, with a 5-year survival rate of 50.8% (2). The global incidence of OC is anticipated to rise by 47% and the number of deaths by 58% by 2045 (3). OC is divided into type I and type II, with type I including Low-Grade Serous Ovarian Carcinoma (LGSOC), clear cell carcinoma, mucinous carcinoma, and endometrioid carcinoma. Type II includes high-grade serous ovarian carcinoma (HGSOC), undifferentiated carcinoma, and malignant mixed mesodermal carcinosarcoma.

First-line treatment for OC includes surgery and platinum- and paclitaxel-based chemotherapy, but approximately 70% of patients relapse and develop platinum and paclitaxel resistance (4). The main mechanisms contributing to chemotherapy resistance in OC include dysregulation of drug transport, alterations of apoptosis, DNA damage repair, alteration of TME, as well as gene mutation and signaling pathway abnormalities (5). Mutations in four genes—Tp53, KRAS, BRCA1/2 and PIK3CA—are strongly associated with the etiology of OC and drug resistance (6).

The Mitogen-Activated Protein Kinases (MAPK) signaling pathway plays a pivotal role in several biological processes, including cell proliferation, differentiation, survival, and stress response (7). It is a fundamental signaling pathway within cells, and its dysregulation is closely associated with the onset and development of OC and the emergence of drug resistance (8). Extracellular Signal-Regulated Kinases (ERK) are extensively researched MAPK signaling pathways that are closely associated with cell growth and developmental divisions. Their transduction pathways adhere to a three-stage enzymatic cascade. In the delivery pathway of ERK, RAS acts as an upstream activating protein, RAF acts as a MAPK Kinase Kinase (MAPKKK), which further activates MEK1/2 through its kinase activity, and MEK1/2 acts as a MAPK Kinase (MAPKK), which can phosphorylate and activate ERK or MAPK. These are activated by adenosine triphosphate (ATP) phosphorylation to form the RAS/RAF/MEK/ERK pathway (9).

A substantial body of evidence indicates that high-frequency mutations in RAS/RAF, which result in aberrant activation of RAS/RAF/MEK/ERK signaling, frequently contribute to drug resistance and poor prognoses in patients with OC (10). Extensive research on the RAS/RAF/MEK/ERK pathway in the context of OC resistance has facilitated the development of numerous MAPK inhibitors, such as Trametinib, Cobimetinib, and Binimetinib. These have been applied to a small subgroup of OC patients to control tumor growth, particularly in LGSOC. For instance, the MEK inhibitor, binimetinib, has been demonstrated to confer clinical benefit to patients with epithelial OC exhibiting MAPK pathway alterations, enhancing the efficacy of paclitaxel-induced apoptosis (11). Additionally, trametinib has emerged as a new standard treatment option for patients with recurrent LGSOC (12).

OC treatment resistance is a complex process involving many molecular mechanisms and signaling pathways. Previous studies have demonstrated that c-Jun N-terminal kinase (JNK) and p38 MAPK impede the progression of OC by regulating apoptosis in response to stressors such as carcinogens, reactive oxygen species (ROS), or oncogenes (13). Fawzy et al. (14) proposed that elevated JNK activity, which contributes to OC chemoresistance, exhibits partial overlap with other pathways, including phosphoinositide 3-kinase (PI3K), nuclear factor-κB (NF-κB), and multiple mechanisms mediating cell transformation and apoptosis.

However, the role of the JNK/p38 MAPK pathway in OC resistance has not been extensively investigated. This review focuses on the mechanism of the JNK/p38 MAPK signaling pathway in OC chemoresistance and provides an overview of potential therapeutic strategies targeting these pathways. The findings will direct researchers’ attention to the mechanism of OC resistance and offer novel research avenues.

## 2 Overview of MAPK signaling pathway

**Figure 1:**
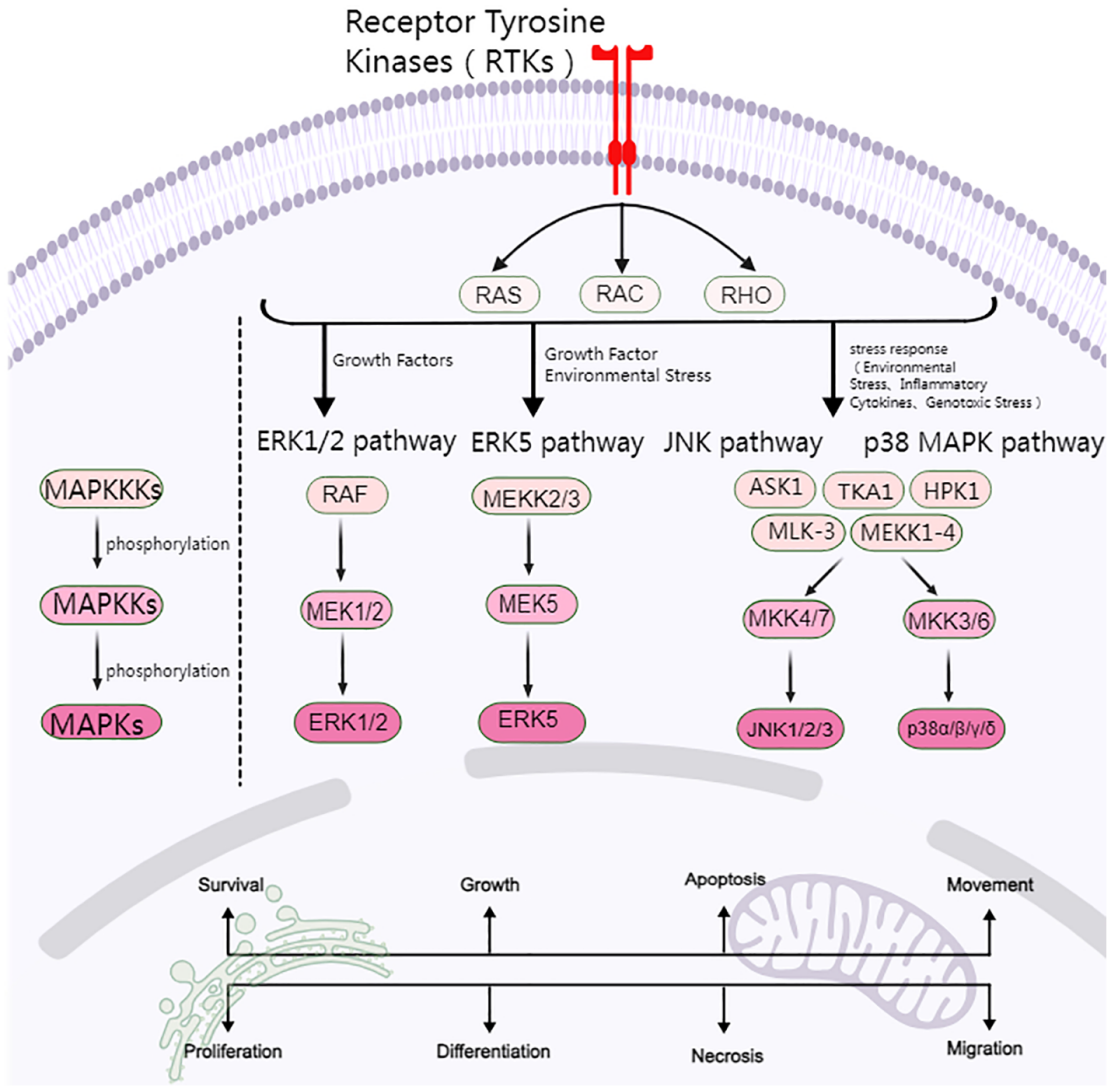
Overview of the MAPK signaling pathway.

MAPK consists of four major cascades: the ERK1/2, ERK5, JNK, and p38 MAPK signaling pathways. The ERK signaling pathway is closely related to cell proliferation and differentiation, while the JNK and p38 MAPK signaling pathways are predominantly linked to cellular stress and apoptosis (**Figure 1**).

### 2.1 ERK signaling pathway

The RAS/RAF/MEK/ERK pathway represents a highly conserved signaling cascade that plays a pivotal role in the survival and development of tumor cells. OC cisplatin resistance is regulated between p53 and RAS signaling networks through RAS/MAPK pathway activation, which mediates apoptosis and autophagy (15). Synuclein is a small neuron protein, and some studies have found that γ-synuclein is significantly upregulated in OC (16). Pan et al. (17) demonstrated that the overexpression of γ-synuclein activated ERK and downregulated JNK, collectively preventing apoptosis. This cell apoptosis is inhibited by 2–3 times, resulting in resistance to paclitaxel and Vincristine chemotherapy. However, the use of MEK1/2 inhibitors has been demonstrated to partially mitigate this resistance to paclitaxel.

Evidence indicates that high-frequency mutations in RAS/RAF result in aberrant RAS/RAF/MEK/ERK signaling activation, leading to platinum-based chemotherapy resistance in OC cells. Manning-Geist et al. found that in type I OC, 60% of LGSOC have alterations in the MAPK pathway (18), with 30–50% and 15–40% carrying RAS and RAF mutations, respectively (19). Additionally, 88% of junctional tumors (20) and 75% of mucinous ovarian tumors also have RAS or RAF mutations (21). Type II tumors almost universally have p53 mutations, and nearly half have BRCA1/BRCA2 mutations (22). Approximately 12% of HGSOC tumors, the most common histological subtype of OC, have been found to have RAS mutations. In OC, particularly in HGSOC, the percentage of RAS gene mutations is generally lower than 10%. However, the transcription of RAF and ERK1/2 can still activate the RAS/RAF/MEK/ERK pathway, which is closely related to the prognosis of OC (23). Consequently, targeting the ERK signaling pathway with RAF, MEK, and ERK inhibitors in OC patients (particularly those with LGSOC) has yielded promising preliminary clinical outcomes compared to the established standard of care (24–27) (**Table 1**).

**Table 1:** Targeting the RAS/RAF/MEK/ERK signaling pathway for OC treatment.

Target	Therapeutic	Cancer Type	Trial Number	Objective Response Rate	Reference
MEK	Binimetinib + paclitaxel	Endometrioid Ovarian Cancer	NCT01849874	14%	(10)
MEK	Binimetinib	LGSOC, fallopian tube or primary peritoneum cancer	NCT01849874	16%	(24)
MEK + PI3K	Binimetinib + buparlisib	Advanced solid tumors	NCT01363232	12%	(25)
MEK	Trametinib	Advanced solid tumor or lymphoma	NCT00687622	10%	(26)
MEK + PI3K	Buparlisib + Trametinib	Advanced solid tumors	NCT01155453	29%	(27)
MEK	Trametinib	LGSOC	NCT02101788	26.2%	(11)

### 2.2 JNK/p38 MAPK signaling pathway

The JNK and p38 MAPK pathways are primarily activated by environmental and genotoxic stressors and regulate cellular responses to various stimuli, including cytokines, inflammatory factors, and growth factors. Consequently, they are often called Stress-Activated Protein Kinases, and they are primarily involved in cellular stress and apoptosis (28).

#### 2.2.1 JNK signaling pathway

The JNK pathway represents a significant branch of the MAPK signaling family, characterized by its capacity to phosphorylate and activate c-Jun transcription factors (29). The JNK pathway is activated by external stimuli, including ultraviolet light, inflammatory factors, and oxidative stress, and regulates the expression of a series of downstream genes. The JNK family comprises three major isoforms: JNK1, JNK2, and JNK3 (30). JNK1 and JNK2 are expressed in most tissues, whereas JNK3 is expressed in a more limited set of tissues, including the brain, heart, and testis (31).

MAPKKKs (e.g., ASK1, HPK1, MLK-3) are the initial kinases to be activated following cell stimulation by stress. These kinases then phosphorylate MAPKKs (MKK4 and MKK7), triggering a signaling cascade (32). MKK4 and MKK7 activate JNK proteins by phosphorylating threonine (Thr183) and tyrosine (Tyr185) at the Thr-Pro-Tyr (TPY) motif of JNK (33, 34). ROS are also involved in JNK activation (33, 34).

Activated JNK protein spreads in the cytoplasm and enters the nucleus. Its phosphorylation leads to the activation or modulation of a range of non-nuclear and nuclear proteins, including c-Jun, Activator Protein-1 (AP-1), and FBJ murine osteosarcoma viral oncogene homolog (FOS). Transcription Factor 2 (ATF-2) and p53, as well as B-cell lymphoma-2 (Bcl-2) and Bcl-2-associated X protein (Bax), are involved in regulating cell proliferation, apoptosis, autophagy, and DNA repair, and they also play a role in influencing OC resistance (35, 36).

#### 2.2.2 p38 MAPK signaling pathway

p38 MAPK is primarily activated by extracellular inflammatory factors, environmental stress, oxidative stress, and DNA-damaging agents (e.g., cisplatin and adriamycin) and is crucial for maintaining cellular homeostasis. The p38 MAPK family comprises four members: p38α, p38β, p38γ, and p38 (35, 36). p38α is universally expressed in most tissues, whereas p38β, p38γ, and p38δ are specifically expressed only in a small number of tissues (37).

Similarly, upstream MAPKKK (TAK1, ASK1, MEKK1–4) is initially activated by dual phosphorylation of Thr and Tyr at the TGY motif, which is then followed by the activation of MAPKKs (MKK3 and MKK6). These MAPKKs subsequently activate p38 by phosphorylating specific serine and Thr residues of p38. It is noteworthy that MKK4 can also phosphorylate p38 MAPK (38). p38 phosphorylates a range of substrates, including the transcription factors ATF2, CHOP, the kinases MK2, MK3, and other effector proteins, which regulate cell cycle progression, cell survival, apoptosis, stress responses, and inflammatory responses. Consequently, p38 plays a role in influencing OC resistance.

JNK/p38 MAPK is often regarded as a critical mediator of cell death and can act as a tumor suppressor (14). However, it can also act as a tumor promoter under certain conditions. Evidence suggests that JNK may play a role in counteracting apoptosis and promoting cancer cell survival under low oxygen or other forms of cellular stress (39). Accordingly, this study will concentrate on the particular mechanisms of JNK and p38 MAPK signaling pathways in drug-resistant OC to offer new insights for future clinical treatment.

## 3 Mechanisms of JNK and p38 MAPK-mediated drug resistance in ovarian cancer

**Figure 2:**
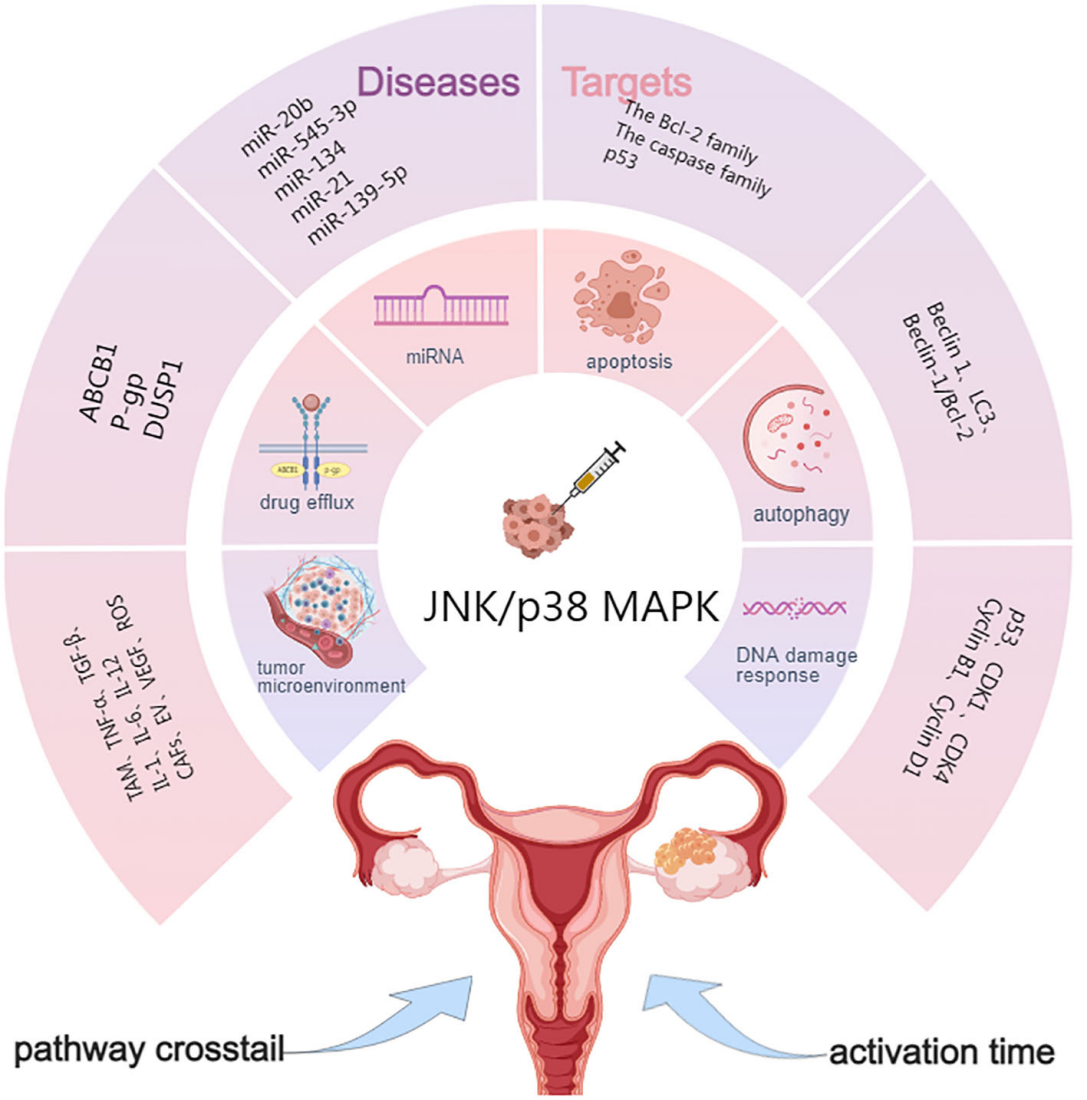
Targets of JNK/p38 MAPK signaling pathway in OC drug resistance. ABCB1, ATP-binding cassette sub-family B member 1; P-gp, P-glycoprotein; DUSP1, Dual specificity phosphatase 1; Beclin 1, LC3, Genes involved in autophagy; TME, Tumor microenvironment; CAFs, Cancer-associated fibroblasts; VEGF, Vascular endothelial growth factor; ECM, Extracellular matrix; EVs, Extracellular vesicles.

Due to the rapid development of chemoresistance, OC remains a significant challenge in oncology. In this section, we summarize the mechanisms involved in JNK/p38 MAPK and OC resistance. These include apoptosis, autophagy, DNA damage response, the tumor microenvironment, drug metabolism, microRNA, activation time, and crosstalk between cell signaling pathways (**Figure 2**).

### 3.1 Apoptosis of cells

Apoptosis, a natural process of programmed cell death, is essential for regulating cellular homeostasis. Dysregulation of apoptosis is a key factor in tumorigenesis and cancer progression. Chemotherapeutic agents exert their anti-tumor effects mainly by inducing apoptosis in cancer cells. The JNK/p38 MAPK signaling pathway modulates the expression and activity of key apoptotic regulators, including the Bcl-2 family, caspase family, and p53, thereby orchestrating critical decisions in apoptotic machinery (37).

#### 3.1.1 The Bcl-2 family

Several factors are responsible for the failure of apoptosis, and the Bcl-2 family, a core factor of apoptosis, is closely associated with chemoresistance in OC (38). The Bcl-2 family is a critical regulator of apoptosis, comprising anti-apoptotic proteins such as Bcl-2, Bcl-extra large (Bcl-xL), and myeloid cell leukemia 1 (Mcl-1), as well as pro-apoptotic proteins including Bcl-2-associated X protein (Bax), Bcl-2-associated death promoter (Bad), and a subgroup of “BH3-only” proteins (e.g., BH3-interacting domain death agonist [Bid], Bcl-2-interacting mediator of cell death [Bim], p53 upregulated modulator of apoptosis [Puma], and phorbol-12-myristate-13-acetate-induced protein 1 [Noxa]). These members orchestrate mitochondrial outer membrane permeabilization through dynamic interactions, ultimately determining cellular fate. Anti-apoptotic proteins prevent apoptosis by stabilizing mitochondrial membrane integrity, while pro-apoptotic proteins promote the increase of mitochondrial outer membrane permeability. JNK/p38 MAPK was shown to induce apoptosis of OC-resistant cells by phosphorylating and inactivating Bcl-2 (39, 40).

BH3-only is a subfamily of the Bcl-2 family, which is considered to promote mitochondrial apoptosis. “BH3-mimetic drugs” inhibit the expression of specific pro-survival Bcl-2 family proteins, promote cell apoptosis, and overcome OC chemotherapy resistance (41). Naftopidil, an α1-adrenergic receptor antagonist, has been shown to overcome resistance to the MEK inhibitor trametinib in OC by activating the JNK signaling pathway and inducing BH3-only protein expression (42). Additionally, arsenic trioxide has been shown to promote apoptosis in OC platinum-resistant cells (43). Yuan et al. (44) found that arsenic trioxide induces apoptosis in OC cells by activating Bim signaling, a member of the BH3-only family, through JNK signaling. Furthermore, they determined the threshold for overcoming cisplatin resistance. Sab is a mitochondrial outer membrane scaffolding protein, and high levels of the protein are associated with decreased Bcl-2 protein and increased BH3-only proteins, thereby restoring the sensitivity of OC cells to chemotherapeutic agents (45). Further studies found that subchronic JNK inhibition induces changes in the concentration of Bcl-2 and Bim proteins by altering Sab expression, leading to increased OC resistance.

#### 3.1.2 The caspase family

The cysteinyl aspartate-specific proteinase (caspase) family also plays a central role in the executive phase of apoptosis. Caspases can also reverse chemotherapy resistance by cutting specific proteins, leading to the destruction of cell structure and function and triggering endogenous or exogenous cell death events. Among them, caspase-3 is a central effector of apoptosis (46). Pan et al. (17) found that overexpression of γ-synuclein can inhibit the activation of the JNK pathway, reduce the phosphorylation of downstream caspase-3, prevent cell apoptosis, and lead to chemotherapy resistance to paclitaxel and vinblastine. Yan et al. (47) showed that prostaglandin could regulate JNK activity and prevent OC cell apoptosis by reducing caspase-3 activity. MT-4, a derivative of mescalin, has been shown to activate p38 MAPK phosphorylation, reduce the expression of heat shock protein 27, and promote the expression of caspase-3 activity, thereby overcoming OC resistance (48). Tang et al. (49) found that cross-reactive material 197 (CRM197), a specific HB-EGF inhibitor, could enhance caspase-3 activity and OC apoptosis via the JNK/p38 MAPK pathway and reverse paclitaxel resistance.

#### 3.1.3 p53

JNK can also promote apoptosis by regulating p53, which is a transcription factor involved in apoptosis, cycle regulation, DNA repair, and other processes (50). The p53 protein plays a key role in Bcl-mediated apoptosis by regulating the pro-apoptotic BH3-only proteins PUMA and NOXA to induce apoptosis (38). It can also prevent the binding of Bcl-2 to pro-apoptotic proteins such as BAX, thereby deregulating the anti-apoptotic function of Bcl-2 and promoting apoptosis (38). Yuan et al. (51) found that PUMA, a p53-upregulated regulator of apoptosis, can sensitize SKOV3 cells to chemotherapy by enhancing caspase activation and downregulating Bcl-xL and Mcl-1, resulting in apoptosis. Studies have shown that p53 mutations lose their cancer suppressor function and promote tumor initiation and progression (52). Potapova et al. (53) demonstrated that inhibiting the expression of JNK1/2 using highly specific JNK antisense oligonucleotides suppressed the growth of cells with mutated p53 (e.g., HGSOC) but did not affect the growth of cells with normal p53 function. This suggests that JNK exerts its pro-apoptotic function only in P53-deficient tumor cells. Using *in vitro* studies, Zhao et al. (54) demonstrated that insulin and cisplatin can activate the JNK signaling pathway, increase p53 expression and the percentage of S-phase cells, promote apoptosis, and enhance the efficacy of cisplatin (**Table 2**).

**Table 2:** Substances affecting OC resistance through apoptosis.

Drug or substance	Effect on JNK/p38 MAPK	Target	Effect on OC	References
Naftopidil	Activate	BH3-ONLY	Overcoming drug resistance	(42)
Arsenic Trioxide	Activate	BH3-ONLY	Overcoming drug resistance	(43, 44)
γ-synuclein	Inhibit	caspase-3	Drug resistance	(17)
Prostaglandin	Inhibit	caspase-3	Drug resistance	(47)
MT-4	Activate	caspase-3	Overcoming drug resistance	(48)
CRM197	Activate	caspase-3	Overcoming drug resistance	(49)
Insulin	Activate	P53	Overcoming drug resistance	(54)

### 3.2 Autophagy

Autophagy is a cellular process of self-degradation and self-protection. Under stress conditions such as chemotherapy, hypoxia, and radiation, autophagosomes are formed to encapsulate damaged organelles and misfolded proteins in the cytoplasm and transport them to lysosomes for degradation, thereby maintaining cellular homeostasis (55). Therefore, autophagy is activated when OC cells are stimulated by chemotherapy drugs (56). Autophagosome formation and increased autophagy flux enable tumor cells to clear the cell damage caused by chemotherapy drugs so that these cells can survive under drug pressure, leading to the generation of drug resistance (57). However, excessive or sustained autophagy can directly mediate or contribute to cell death and reverse OC resistance (57, 58).

When cells are exposed to commonly used chemotherapy drugs such as cisplatin or paclitaxel, JNK/p38 MAPK activates and regulates the expression of autophagy-associated proteins, promoting autophagosome formation and enhancing autophagy flow. Beclin 1 and Microtubule-associated protein 1 light chain 3 (LC3) are crucial in autophagy. The JNK/p38 MAPK signaling pathway induces autophagy in OC cells and increases autophagosome formation and autophagic flux by promoting the conversion of LC3-I to LC3-II and the formation of autophagosomes (59). Zhu (60) et al. analyzed the mutation profiles of 62 HGSOC and found that overexpression of FGF19 in the fibroblast growth factor family promotes the phosphorylation of p38 to induce autophagy, upregulates the expression of LC3 and Beclin-1, and promotes cisplatin resistance in OC cells. Conversely, the knockdown of FGF19 downregulates the phosphorylation of p38 to reverse cisplatin chemoresistance.

The Unfolded Protein Response (UPR) is a pro-survival mechanism that is activated when unfolded or misfolded proteins accumulate in the endoplasmic reticulum (ER), leading to JNK/Ap-1 activation and increased autophagy (61). Yang et al. (62) found that JNK3 activation through the UPR pathway promoted acid/lysosomal compartment accumulation, blocked autophagy flux during ER stress, and reduced ROS levels in OC-resistant cells to promote OC cell survival.

Sustained activation of the JNK/p38 MAPK pathway can lead to apoptosis or excessive autophagy, resulting in a state of “autophagy cell death” or “autophagy addiction.” JNK/p38 MAPK can also reverse OC resistance by regulating the balance between apoptosis and autophagy (63). Zhao et al. (64) found that propranolol can upregulate the expression of LC3-II and caspase-3 by activating the ROS/JNK signaling pathway, induce apoptosis and autophagy of OC cells, and restore cell sensitivity to chemotherapy. Guo et al. (65) demonstrated that MAP2K6-FP, a genetically engineered fusion protein targeting the MAPK kinase pathway, enhances paclitaxel sensitivity in OC by inducing autophagy. This chimeric protein contains three functional domains (1): a HO8910 OC cell-specific binding peptide (2), the TAT protein transduction domain for cellular internalization, and (3) the MKK6(E) effector domain with anti-tumor activity. Mechanistically, MAP2K6-FP upregulated p38 expression, elevated LC-3 and Beclin-1/Bcl-2 levels, and amplified autophagic flux, thereby overcoming chemoresistance in OC models. Mishra et al. (66) showed that photothermal therapy can induce autophagy to overcome drug resistance by activating JNK signaling and UPR signaling pathways in OC chemoresistant cells.

While autophagy serves as a self-protection mechanism that enables resistance of OC cells to chemotherapy, sustained activation of autophagy by JNK/p38 MAPK signaling can trigger the “autophagic cell death process.” Autophagic cell death can override cellular survival mechanisms by excessive autophagy or modulating the balance between apoptosis and autophagy. This process re-sensitizes OC cells to chemotherapeutic agents and reverses OC drug resistance.

### 3.3 DNA damage response

Platinum chemotherapeutic agents, such as cisplatin and carboplatin, impede DNA replication and transcription by twisting the DNA Double helix, causing single-strand breaks, double-strand breaks (DSB), and chromosome rearrangements, ultimately resulting in cell death. When DNA damage occurs, abnormal activation of the DNA damage response (DDR) is responsible for pausing the cell cycle and initiating DNA repair. Cell cycle checkpoints regulate cell cycle transitions by acting on the cell’s G1, S, and G2/M phases. This process can provide time for DNA repair when cell cycle arrest occurs, leading to chemotherapy resistance (67). However, in some cases, inducing cell cycle arrest at specific stages and increasing the accumulation of DNA damage can promote apoptosis and reverse chemoresistance in OC cells.

#### 3.3.1 DNA damage repair

JNK/p38 MAPK is activated when stimulated by chemotherapeutic agents to regulate apoptosis and enhance the effect of chemotherapeutic agents. However, in DDR, JNK/P38 MAPK can be activated in response to DNA damage, participating in the DNA damage repair process and leading to treatment resistance (68, 69). Cell division cycle 2 (Cdc2)-like kinase plays a role in RNA splicing, cell cycle regulation, DDR, and other cellular functions. Jiang et al. (70) found that p38 can enhance DNA damage repair through phosphorylation of Cdc2-like kinase, resulting in OC resistance. Erlotinib exerts its anti-tumor effects by inducing DSBs in cancer cells, triggering cell death by the accumulation of unrepaired DNA damage (71). However, p38 MAPK activation can initiate the DDR mechanism, which repairs the DNA damage and regulates cell cycle checkpoints. This repair process reduces DNA damage-induced apoptosis, ultimately contributing to erlotinib resistance (72). In p53-deficient tumor cells, the p38/MK2 pathway is activated and reactivates cell cycle checkpoints to repair the damage, thereby inducing chemotherapy resistance (73, 74). However, when combined with platinum or paclitaxel, RNA peptide nano complexes blocked cell cycle inspection of p38/MK2, sensitized pp53 deficient HGSOC mice to chemotherapeutic agents, and improved overall survival by 37% (75). Seino et al. (76) demonstrated that the high activity of JNK would promote cisplatin resistance through various phosphokinase assays, and the combination of cisplatin and JNK inhibitor (SP600125) could enhance DNA damage to reverse cisplatin resistance. The longevity gene SIRT6 has been identified as a critical factor in stimulating DSB repair. Studies have shown that JNK activates DNA DSB repair by stimulating the phosphorylation of SIRT6 during oxidative stress conditions, leading to OC resistance (77).

#### 3.3.2 Mechanisms of cell cycle regulation

JNK/p38 MAPK can reverse chemoresistance by inducing cell cycle arrest and amplifying DNA damage accumulation. Cyclin D1 and CDK4/6, critical regulators of the G1/S transition, form complexes that drive cell cycle progression (78). However, p38 MAPK inhibits this process by degrading Cyclin D1 and disrupting Cyclin D1/CDK4 complexes, thereby arresting cells in the G1/S phase (79). Cannell et al. (80) demonstrated that p38 could bind to growth arrest and DNA damage-inducible 45α (Gadd45α), control G1/S and G2/M cell cycle checkpoints, and influence OC resistance. Similarly, tocotrienols activate JNK/p38 MAPK signaling to induce G1/S arrest, leading to OC cell death and re-sensitization to cisplatin (81).

In the G2/M phase, Cyclin B1/CDK1 complexes promote cell cycle progression (82), but p38 MAPK exerts checkpoint control by degrading Cyclin B1. This regulatory mechanism is exploited by therapeutic agents such as Chaetoglobosin K (ChK), a fungal metabolite that induces G2 arrest through p53 activation and p38-dependent Cyclin B1 phosphorylation, selectively inhibiting cisplatin-resistant OC cells (83). Conversely, cell cycle and apoptosis regulator 2 promote cisplatin resistance by suppressing p38 signaling and sustaining Cyclin B1/CDK1 activity (84).

Notably, pharmacological modulation of this checkpoint demonstrates clinical potential. The synthetic compound MPT0G066 (13.28-fold more potent than paclitaxel) activates JNK to induce G2/M arrest, thereby re-sensitizing resistant OC cells to cisplatin (85). LB100, a PP2A inhibitor, eliminates cisplatin-induced G2/M arrest by prolonging JNK activation, effectively overriding checkpoint-mediated survival and restoring chemosensitivity (86).

### 3.4 The tumor microenvironment

TME consists of immune cells, Cancer-associated Fibroblasts (CAFs), Vascular Endothelial Growth Factor (VEGF), adipocytes, Extracellular Matrix (ECM), and extracellular vesicles (EVs), which play a crucial role in OC treatment resistance. Due to the unique characteristics of OC metastasis in the peritoneal cavity, OC cells can survive in ascites as floating unicellular or multicellular spheres, forming a characteristic OC TME (87). Increasing evidence suggests that targeting the TME may be a promising strategy for reversing OC chemoresistance.

Tumor-associated macrophages, an essential component of immune cells in TME, can secrete many pro-inflammatory cytokines, TNF-α, TGF-β, IL-1, IL-6, and IL-12, to enhance the immune response. JNK can be activated by TGF-β, interferon-γ, and other mediated immune evasion mechanisms that affect OC resistance (76, 88). Mitra et al. (89) found that TGFβ1 can induce stemness and chemoresistance in OC cells by activating ERK1/2 and JNK/p38 MAPK pathways and targeting them to participate in epithelial-mesenchymal transition (EMT). Bioinformatic analysis of Gene Expression Omnibus and The Cancer Genome Atlas databases revealed that CAFs affect OC chemoresistance through the p53, cell cycle, PI3K-AKT, and MAPK pathways (90). Izar et al. (91) found that stimulating the expression of CAFs stimulated the formation of OC-specific TME, which increased HGSOC aggressiveness and drug resistance. IL-6 secreted by CAFs is one of the essential pro-inflammatory cytokines and plays a crucial role in OC resistance (92). Alspach et al. (93) reported that p38 promotes IL-6 expression by affecting the RNA-binding protein AUF1, but inhibition of p38 blocks the tumor-promoting ability of CAFs. Curtis et al. (94) found that p38 can provide nutrients through glycogen phosphorylation to alter the metabolic state in OC TEM and support the survival of drug-resistant OC cells. Samuel et al. (95) found that administering chemotherapeutic agents to OC cells induced EV release via JNK/p38 MAPK signaling, increasing drug resistance in OC cells. Octreotide, a somatostatin analogue, in combination with paclitaxel, can reverse paclitaxel resistance by inhibiting p38 and decreasing the expression of VEGF (96).

ROS, a critical regulator of TME, is closely related to the occurrence of OC resistance (97). Elevated levels of ROS can induce oxidative DNA damage and stimulate long-term activation of JNK, which plays an essential role in reversing drug resistance (98). ROS can mitigate OC resistance by oxidizing cysteine residues of ASK1, thereby activating the downstream JNK/p38 MAPK pathway (99). Li et al. (100) found that BH3-only protein mimic ABT-737 enhanced the activation of JNK and ASK1 by increasing ROS levels in cells in a time-dependent manner, overcoming cisplatin resistance in A2780/DDP cells. Ovendazole can inhibit the proliferation of drug-resistant OC cells through ROS-mediated activation of the JNK/p38 MAPK signaling pathways, leading to G1/S or G2/M cell cycle arrest (99).

Thus, JNK/p38 MAPK activation interacts with immune cells and pro-inflammatory factors in the TME to support the survival of OC cells, leading to chemoresistance. However, during persistently elevated levels of ROS, sustained activation of JNK/p38 MAPK can lead to apoptosis of otherwise drug-resistant cells. This finding offers insights into the development of novel therapeutic strategies for treating OC resistance.

### 3.5 Drug effluent

Multidrug Resistance (MDR) leads to a lack of sensitivity to multiple chemotherapy drugs, which is the main reason for chemotherapy failure. The overexpression of ATP-binding cassette (ABC) transporters is the leading cause of MDR in chemotherapy. P-glycoprotein (P-gp), the most studied protein of the ABC transporter family, is encoded by the ABCB1 gene encodes and affects the uptake of chemotherapy drugs by pumping them out of tumor cells (101).

Evidence suggests that the p38 signaling pathway can enhance drug efflux and participate in OC resistance formation by inducing the upregulation of ABC transporter protein expression. p38 can increase the expression of ABC transporter proteins by activating the downstream transcription factor NF-κB, which binds to and upregulates the ABCB1 promoter. Afatinib, an ATP-competitive aniline-quinazoline compound, can inhibit ABCB1 transcriptional expression by downregulating p38-dependent activation of NF-κB, thereby reversing ABCB1-mediated MDR (102). Dual specificity phosphatase 1 (DUSP1), also known as MKP-1, is a class of phosphokinase capable of dephosphorylating MAPK substrates. Overexpression of DUSP1 leads to increased phosphorylation of p38. Kang et al. (103) found that activation of p38 by DUSP1 enhanced the expression of P-gp, but did not alter the activation of ERK1/2 and JNK1/2, ultimately affecting the efflux of anticancer drugs from the OC.

### 3.6 MicroRNA

MicroRNA (miRNA) is a class of small non-coding RNAs, which are 18–25 nucleotides long, and alter tumor sensitivity to treatment by regulating gene expression and apoptosis (104). Dysregulation of miRNA expression is an important cause of OC chemotherapy resistance (105). Therefore, correcting miRNA dysregulation is a promising therapeutic strategy to reverse chemoresistance (106).

Inhibition of the JNK/p38 MAPK signaling pathway was found to downregulate the expression levels of miRNAs involved in regulating chemotherapeutic drug sensitivity-related miRNAs and mediated OC chemoresistance. Kumar et al. (107) showed that the downregulation of miR-20b targets genes such as DUSP8 and inhibits p38 in A2780/CP70 drug-resistant OC cell lines, leading to cisplatin resistance. Yin et al. (108) showed that downregulating miR-545-3p leads to cisplatin resistance by reducing the activity of the JNK signaling pathway. Fos-related antigen-1 (Fra-1), a part of the AP-1 transcription factor complex, can amplify ERK/JNK signaling and reduce chemosensitivity in OC cells by promoting miR-134 expression (109). Activation of JNK-1/c-Jun pathway was found to upregulate its miR-21 expression, leading to decreased levels of the tumor suppressor gene programmed cell death protein 4 (PDCD4), increased cell proliferation, and development of OC cisplatin resistance (110). Jiang et al. (111) showed that restoring the expression of miR-139-5p in drug-resistant cells can inhibit the expression of c-Jun, increase the expression of Bcl-xl, activate caspase-9 and caspase-3, and reverse cisplatin resistance in OC (**Table 3**).

**Table 3:** Effect of miRNAs on OC drug resistance.

miRNA	Target	Signaling pathways	Function	Impact on OC	References
miR-20b	DUSP8	p38	Downregulate	drug-resistant	(107)
miR-545-3p	PPA1	JNK	Downregulate	drug-resistant	(108)
miR-134	Fra-1	ERK/JNK	Upregulate	drug-resistant	(109)
miR-21	PDCD4	JNK	Upregulate	drug-resistant	(110)
miR-139-5p	Bcl-xl/caspase-3/9	JNK	Downregulate	reverse drug resistance	(111)

### 3.7 Activation time

Further study of the JNK/p38 MAPK pathway has revealed that different activation times may lead to different cellular responses and biological effects. The JNK/p38 MAPK pathway is usually activated during the initial phase of chemotherapy to induce apoptosis or other cell death mechanisms. However, in drug-resistant cells, activation of the pathway may be delayed, attenuated, or shortened in duration.

Different activation timings may determine the fate of OC cells. Transient JNK/p38 MAPK activation is usually associated with pro-survival effects, whereas sustained JNK/p38 MAPK activation may be pro-apoptotic (112). Davis et al. (112) found that the activation time of JNK/p38 MAPK persisted for 12 hours in cisplatin-sensitive cells, while it was limited to 1–3 hours in cisplatin-resistant cells. LB100 can eliminate cisplatin-induced cell cycle arrest and sensitize OC cells to cisplatin by altering the temporal order and persistence of JNK activation (91). Elevated expression of HB-EGF (an EGFR ligand) contributes to cancer cell resistance to paclitaxel, and shedding the extracellular domain of HB-EGF can induce sustained activation of JNK/p38 MAPK to overcome this resistance (113). Mansouri et al. (114) showed that the JNK/p38 MAPK pathway plays a central role in cisplatin-induced apoptosis of OC cells by activating the expression of the downstream target pro-apoptotic cytokine FasL. Furthermore, the duration of sustained activation is a critical early determinant of cisplatin-induced apoptosis, as transient activation fails to maintain c-Jun and ATF-2 phosphorylation and upregulate FasL expression.

Thus, successful treatment depends not only on the combination and dosage of drugs but also on the timing, duration, and order of administration (115). A better understanding of the dynamic process of OC resistance will facilitate the clinical development of more effective therapeutic strategies for OC resistance.

### 3.8 Crosstalk between signaling pathways

The mechanism of drug resistance in OC is complex and multifactorial, involving multiple pathways. The PI3K/AKT pathway engages in bidirectional crosstalk with JNK/p38 MAPK, creating a dynamic equilibrium that dictates OC cell survival under therapeutic stress. Mechanistically, AKT suppresses JNK-mediated apoptosis by phosphorylating and inactivating ASK1, a key upstream kinase in the JNK pathway (116). Conversely, p38 inhibition prevents AKT dephosphorylation and inhibits the AKT pathway, thereby reinforcing PI3K/AKT-dependent survival signaling—a paradoxical interaction that underscores the complexity of pathway interdependencies (117). This stress-adaptation mechanism contributes to the development of OC resistance. For example, the ER protein HERPUD1 activates both PI3K/AKT/mTOR and p38 pathways to maintain autophagy and block apoptosis, thereby driving platinum resistance in OC models (118). Clinically, compensatory pathway activation poses a significant challenge. Resistance to PI3K/AKT inhibitors in OC patients is frequently associated with ERK upregulation and p38 suppression, suggesting a survival mechanism where ERK compensates for inhibited AKT. Notably, dual inhibition of ERK and AKT disrupts this cytoprotective feedback, synergistically eliminating cisplatin-resistant OC cells (119, 120). Growth factor signaling further amplifies this network, with FGF2 overexpression activating p38 and AKT to upregulate anti-apoptotic proteins (Bcl-2/Bcl-xL) and cell cycle drivers (Cyclin D1). Co-targeting FGF2-activated p38 and PI3K/AKT pathways reverses adriamycin resistance, highlighting the therapeutic potential of combinatorial approaches (121).

NF-κB acts as a key sink node to promote inflammatory responses and cellular survival under stress by blocking DNA damage-induced apoptosis through the inhibition of sustained activation of JNK (122, 123). Meanwhile, p38 upregulates anti-apoptotic proteins (such as Bcl-xL) and inflammatory factors (such as IL-6) through NF-κB activation. Hence, p38 promotes OC cell survival (124, 125).

Additionally, Wnt/β-catenin and HIF-1α pathways are involved in JNK/p38MAPK pathway crosstalk. Phosphorylation of β-catenin by JNK contributes to OC chemotherapy resistance by promoting cancer stem cell survival and inducing EMT (126–128). Furthermore, glycolysis mediated by the p38-HIF1α axis promotes the survival of chemoresistant OC cells in a hypoxic environment. Modulating HIF-1α activity by inhibiting p38, in combination with glucose analogues and platinum compounds, could enhance the efficacy of chemotherapy and represents a promising therapeutic strategy for OC (129).

In summary, the intricate crosstalk between JNK/p38 MAPK, PI3K/AKT, NF-κB, Wnt/β-catenin, and metabolic pathways forms a robust network that sustains OC cell survival under therapeutic stress. While this complexity poses significant challenges, it also offers multiple therapeutic vulnerabilities. Future research should focus on combinatorial therapies: Simultaneously targeting multiple nodes (e.g., PI3K/AKT + JNK/p38 + NF-κB) to overcome compensatory pathway activation.

## 4 JNK/p38 MAPK inhibitors and natural compounds to treat OC resistance

Targeting JNK and p38 MAPK may be a promising strategy to reverse OC resistance due to their important roles in chemoresistance. Several potent and specific inhibitors of JNK and p38 MAPK have been developed to restore cancer cells’ sensitivity to chemotherapy drugs and reverse chemotherapy resistance (**Table 4**).

**Table 4:** Summary of inhibitors targeting the JNK/p38 MAPK signaling pathway.

Target	Name of inhibitor	Mode of action	Note	References
JNK	SP600125	Regulate G2/M phase	Non-specific: may inhibit multiple kinases	(122)
		Enhanced DNA damage	Therapeutic effect attenuated when combined with paclitaxel, but pretreatment of OC cells for three days enhanced the sensitivity of cisplatin and paclitaxel	(76)
JNK	AS602801	Decreases survivin expression	Not effective against carboplatin or cisplatin	(123)
p38 MAPK	SB203580	Increase apoptosis	Non-specific: affecting other members of the MAPK family	(130)
		Increased ERCC1 expression		(131)
JNK/p38 MAPK	SB203580/SP600125	Suppresses EMT		(132)
JNK + p38 MAPK	SB202190 + SP600125	Inhibits autophagic flux and EMT		(133)
p38 MAPK	BIRB796	Inhibits ABCB1-mediated MDR	Entered clinical trials (NCT02211157)	(134)

### 4.1 JNK inhibitors

SP600125 is a JNK inhibitor that inhibits JNK kinase activity and the JNK signaling pathway by specifically blocking the ATP-binding site of the JNK protein. There is growing evidence that SP600125 can mediate autophagy and apoptosis to overcome drug resistance in several malignant tumors and reverse OC resistance by regulating the cell cycle in the G2/M phase (122). Seino et al. (76) showed that combining cisplatin and SP600125 enhanced DNA damage to reverse cisplatin resistance, but the therapeutic effect was diminished when SP600125 was combined with paclitaxel. Notably, pretreating OC cells with SP600125 for three days before administrating cisplatin or paclitaxel inhibited basal JNK activity and reduced chemotherapy resistance without increasing toxicity. Thus, this time-staggered inhibition of JNK effectively enhances the sensitivity of cisplatin and paclitaxel. However, the clinical application of SP600125 is limited because it is a broad-spectrum JNK reversible inhibitor that is non-specific and can inhibit multiple kinases. AS602801 is an oral, selective JNK inhibitor and anticancer stem cell candidate, which has been shown to reduce the expression of survivin, making OC-resistant cells sensitive to paclitaxel but not carboplatin or cisplatin (123). In the future, AS602801 can be combined with other inhibitors to inhibit the growth and survival of OC cells more effectively.

### 4.2 p38 inhibitor

Evidence suggests that p38 inhibitors can increase the sensitivity of OC cells to cisplatin and reverse drug resistance. SB203580, a widely used p38 inhibitor, showed a synergistic effect of cisplatin and SB203580 in OC Cisplatin IGROV-1/Pt1 cells, resulting in greater apoptosis than cisplatin alone (130). Xie et al. (131) found that metformin combined with SB203580 application inhibited Excision Repair Cross-Complementation Group 1 (ERCC1) expression in OC-resistant cells and enhanced the sensitivity of OC cisplatin-resistant cells. Additionally, JNK inhibitors can be used in combination with p38 inhibitors. Zhu et al. (132) found that SB203580 or SP600125 inhibited EMT and reduced metastasis in OC cells. Chen et al. (133) showed that SB202190 combined with SP600125 inhibited autophagy flux and EMT to inhibit drug resistance. Despite the promising evidence from *in vivo* and *in vitro* studies demonstrating the effectiveness of JNK/p38 MAPK inhibitors, progress has been slow in clinical trials. The p38 inhibitor BIRB 796 has been shown to inhibit ABCB1-mediated MDR147 and is currently undergoing clinical trials (NCT02211157) (134).

Research on JNK/p38 MAPK inhibitors in OC is still in the initial stage. JNK/p38 MAPK inhibitors may cause side effects on normal cells, affecting their efficacy and safety *in vivo*. Additionally, many inhibitors lack specificity and may interact by targeting other kinases, affecting drug selectivity and side effects. Furthermore, effective drug delivery remains a crucial challenge because different isoforms have different functions in a cellular environment-dependent manner. Further in-depth studies on its mechanism of action and clinical application are needed to develop new strategies and methods for treating OC.

### 4.3 Natural compounds targeting JNK/p38 MAPK to reverse ovarian cancer chemoresistance

Natural compounds derived from medicinal plants and traditional formulations have emerged as promising agents to overcome chemoresistance in OC by modulating JNK/p38 MAPK signaling. Compared with traditional chemotherapy, these compounds exhibit multi-target effects, including apoptosis induction, DNA repair suppression, and cell cycle arrest, while demonstrating lower systemic toxicity than conventional chemotherapy (135) (**Table 5**).

**Table 5:** Natural compounds reverse OC resistance via JNK/p38 MAPK.

Category	Compound	Source	Pathway	Target	Function	Reference
Apoptotic	Astragalus polysaccharide	Astragalus membranaceus	JNK	Bcl-2	Downregulate	(136)
	Tubeimoside I	Bolbostemma paniculatum	p38	Bcl-2	Downregulate	(137)
	Deguelin	Derris trifoliata	p38	Bcl-2/Mcl-1	Downregulate	(40)
	Kaempferol	Fruits/vegetables	ERK/JNK/CHOP	DR4/DR5	Upregulate	(138)
	Noscapine	Papaver somniferum	JNK	Disrupt tubulin dynamics		(139)
DNA Repair Disruption	Glaucocalyxin B	Rabdosia japonica	JNK	DNA repair disruption		(140)
	β-Elemene	Curcuma wenyujin rhizomes	JNK	DNA repair disruption		(141)
	Tanshinone IIA	Salvia miltiorrhiza	p38	ERCC1	Downregulate	(142)
Cell Cycle Arrest	NK007	Litsea cubeba (Lauraceae)	p38	G1/S		(143)
	Curcumin	Curcuma longa	p38 PI3K/AKT	G2/M		(144)
	Evodiamine	Evodia officinalis	p38	G2/M P-gp	Downregulate	(145)

#### 4.3.1 Apoptotic pathway activation

Astragalus polysaccharide, derived from *Astragalus membranaceus*, activates JNK to downregulate Bcl-2 and upregulate Bax/caspase-3, restoring cisplatin sensitivity in OC cells (136). Tubeimoside I, derived from *Bolbostemma paniculatum*, enhances p38-mediated Bax activation while suppressing Bcl-2, effectively reversing platinum resistance (137). Deguelin, a flavonoid from *Derris trifoliata*, synergizes with paclitaxel to inhibit p38, downregulating Bcl-2 and Mcl-1 in SKOV3-TR cells (40). Kaempferol, a flavonoid from fruits/vegetables, upregulates ERK/JNK/CHOP signaling and increases death receptors (DR4/DR5) expression, overcoming OC resistance (138). Noscapine, derived from *Papaver somniferum*, activates JNK to induce apoptosis in paclitaxel-resistant cells by disrupting tubulin dynamics (139).

#### 4.3.2 DNA repair disruption

Glaucocalyxin B, derived from *Rabdosia japonica*, overcomes OC cisplatin resistance by mediating JNK pathway activation and increasing DNA damage (140). β-Elemene, derived from *Curcuma wenyujin rhizomes*, downregulates DNA repair activity and inhibits JNK activation, sensitizing OC cells to cisplatin (141). Tanshinone IIA, derived from *Salvia miltiorrhiza*, activates p38 to downregulate ERCC1, a critical mediator of platinum adduct removal (142).

#### 4.3.3 Cell cycle arrest

NK007, derived from *Litsea cubeba*, induces G1/S arrest via p38 upregulation, overcoming paclitaxel resistance (143). Curcumin, derived from *Curcuma longa*, induces p38-dependent G2/M arrest and inhibits PI3K/AKT survival signaling in cisplatin-resistant OC cells (144). Evodiamine, derived from *Nvidia officinal*, induces G2/M phase arrest by activating p38 and inhibits P-glycoprotein (P-gp)-mediated drug efflux (145).

#### 4.3.4 Clinical challenges and emerging strategies

In treating OC, natural compounds have demonstrated significant potential for reversing chemoresistance by modulating the JNK/p38 MAPK signaling pathway. However, clinical application faces challenges, including poor bioavailability, limited pharmacokinetic stability, and batch-to-batch variability in quality control.

Advanced delivery systems have been developed to improve efficacy and reduce off-target toxicity. For instance, triptolide-loaded nanoparticles derived from *Tripterygium wilfordii* enhance the targeting efficiency of JNK/p38 while reducing hepatotoxicity (146). Similarly, curcumin demonstrates promising effects in drug-resistant human OC cells when delivered via nanocarriers (146). These innovative approaches improve the therapeutic efficacy of natural compounds and mitigate their side effects, enhancing their clinical applicability. Future studies on bioavailability, pharmacokinetics, and quality control of natural compounds are expected to change the treatment landscape for patients with OC resistance.

## 5 Discussion and conclusion

The JNK/p38 MAPK signaling pathway exhibits a dual role in OC chemoresistance, paradoxically promoting both apoptosis and tumor cell survival. Our analysis reveals that JNK/p38 MAPK activation under chemotherapy-induced stress initially triggers apoptosis and DDR to eliminate compromised cells. However, dysregulated signaling shifts toward pro-survival mechanisms, including enhanced autophagy, DNA repair, and drug efflux, enabling OC cells to evade treatment. This adaptive reprogramming is amplified through crosstalk with PI3K/AKT, NF-κB, Wnt/β-catenin, and HIF-1α pathways, collectively sustaining a therapy-resistant TME. Notably, transient JNK/p38 MAPK activation promotes survival, whereas prolonged signaling induces apoptosis or autophagic cell death. This temporal duality highlights the potential of chronotherapeutic approaches, such as staggered chemotherapy dosing, to exploit cumulative DNA damage in resistant cells.

Targeting JNK/p38 MAPK presents a promising strategy to reverse OC chemoresistance. Preclinical studies demonstrate that inhibitors like SP600125 (JNK) and SB203580 (p38) restore cisplatin sensitivity by augmenting apoptosis and cell cycle arrest, although clinical translation remains limited. Natural compounds, including curcumin, Astragalus polysaccharides, and β-elemene, show multimodal regulation of JNK/p38 MAPK signaling with lower toxicity profiles compared to conventional chemotherapeutics. Nanotechnology innovations (e.g., curcumin-loaded liposomes and triptolide nanoparticles) address bioavailability challenges while improving tumor-specific delivery.

Despite these advances, clinical implementation faces multifaceted challenges. First-generation JNK/p38 MAPK inhibitors exhibit off-target effects and systemic toxicity. While natural products offer multi-target advantages, standardization issues related to pharmacokinetics, stability, and quality control hinder their clinical adoption. Furthermore, OC heterogeneity necessitates biomarker discovery (e.g., p38α overexpression, RAS/RAF mutations) to identify patients most likely to benefit from JNK/p38-targeted therapies.

Future research should focus on the following three aspects. First, exploring the mechanism of action of the JNK/p38 MAPK signaling pathway, developing JNK and p38 MAPK inhibitors with higher specificity and fewer adverse reactions, and exploring new natural compounds will be key to advancing OC treatment. Second, combination therapy targeting multiple nodes in the JNK/p38 MAPK pathway and other interacting pathways helps to overcome compensatory pathway activation. Alternatively, interleaved administration before chemotherapy, combined with a chronotherapy approach, provides a more comprehensive therapeutic strategy. Third, advancing research and the discovery of innovative biomarkers such as p38α overexpression and RAS/RAF mutations will facilitate the stratification of patients according to their gene expression profiles, thereby providing personalized treatment options for patients. Therefore, regulating JNK/p38 MAPK can help mitigate resistance to OC chemotherapy ultimately improving the survival and quality of life of OC patients.

## Glossary

AKT: protein kinase B
AMPK: AMP-activated protein kinase
CAF: Cancer-associated fibroblast
OC: Ovarian cancer
MAPK: Mitogen-Activated Protein Kinases
ERK: Extracellular Signal-Regulated Kinases
JNK: c-Jun N-terminal kinase
LGSOC: Low-Grade Serous Ovarian Carcinoma
HGSOC: High-grade serous ovarian carcinoma
ROS: Reactive oxygen species
NF-κB: Nuclear factor-κB
PI3K: Phosphoinositide 3-kinase
TME: Tumor microenvironment
TNF-α: Tumor necrosis factor-α
TGF-β: Transforming growth factor-β
IL-1: Interleukin-1
IL-6: Interleukin-6
IL-12: Interleukin-12
VEGF: Vascular endothelial growth factor
ECM: Extracellular matrix
EVs: Extracellular vesicles
MDR: Multidrug resistance
ABC: ATP-binding cassette
P-gp: P-glycoprotein
DUSP1: Dual specificity phosphatase 1
MKP-1: MAPK phosphatase-1
miRNA: MicroRNA
DSB: Double-strand breaks
DDR: DNA damage response
Gadd45α: Growth arrest and DNA damage-inducible 45α
ChK: Chaetoglobosin K
HB-EGF: Hepatocyte growth factor-regulated tyrosine kinase substrate
FGF2: Fibroblast growth factor 2
HIF-1α: Hypoxia-inducible factor-1α
ERCC1: Excision repair cross-complementation group 1
